# Seasonal Shifts from Water Depth to Nitrate Reorganize Protistan Communities Following Lake Freeze–Thaw Events

**DOI:** 10.3390/microorganisms13122869

**Published:** 2025-12-17

**Authors:** Yanying Zhou, Zhengming Luo, Jinxian Liu

**Affiliations:** 1 Department of Biology, Xinzhou Normal University, Xinzhou 034000, China; 18135051862@163.com (Y.Z.); luozhengming2004@126.com (Z.L.); 2 Shanxi Key Laboratory of Ecological Restoration for Loess Plateau, Field Scientific Observation and Research Station of the Ministry of Education of Shanxi Subalpine Grassland Ecosystem, Institute of Loess Plateau, Shanxi University, Taiyuan 030006, China

**Keywords:** protist, freezing and thawing, diversity, mountain lake

## Abstract

The seasonal freeze–thaw cycle induces a fundamental regime shift in lake ecosystems, primarily through the restructuring of microbial communities. This study investigated the dynamics and mechanisms of species diversity maintenance in protistan communities in Gonghai Lake, a shallow subalpine lake in China, across both ice-covered and ice-free periods. During ice cover, the protistan community exhibited a vertically stratified structure dominated by cryotolerant diatoms such as *Stephanodiscus*. Following thaw, the community transitioned to a more homogeneous, resource-driven assembly. Concurrently, the key environmental controls shifted from physical stratification (proxied by depth) to resource availability (notably NO_3_^−^ and TOC), a change reflected in the taxonomic succession from Ochrophyta to Chlorophyta. Nevertheless, depth retained ecological relevance mediated by benthic–pelagic coupling, which supported a distinct near-sediment community. Our findings demonstrate that freeze–thaw-mediated terrestrial nutrient inputs directly modified protistan diversity and community structure. These alterations have fundamental implications for ecosystem functions in subalpine lakes, including nutrient cycling rates and energy flow through the microbial loop.

## 1. Introduction

Seasonal ice cover characterized lakes in boreal and temperate regions, where the freeze–thaw process imposed considerable seasonal variations on lake ecosystems [1]. Against the backdrop of a globally reduced ice-cover duration driven by climate change, understanding the community dynamics in aquatic ecosystems experiencing freeze–thaw cycles has become increasingly urgent [2]. The annual ice phenology not only directly alters the physical environment (e.g., light penetration and water column stratification) but also triggers a cascade of limnological changes that fundamentally reshape the habitat for aquatic organisms [3,4]. During the ice-covered period, the lake is isolated from atmospheric exchange, often leading to hypoxia or anoxia in the underlying water, while simultaneously inhibiting wind-driven turbulence [5]. These conditions favor the accumulation of dissolved organic carbon and nutrients from microbial degradation and sediment release [6]. The subsequent ice-off and spring thaw induce abrupt shifts: sudden re-oxygenation, rapid water column mixing, increased light availability, and the potential pulsed release of accumulated nutrients and dissolved organic carbon [7,8]. These dramatic transitions create a strong environmental filter and temporal niche opportunities, placing unique selective pressures on the lake’s biological communities.

Protists, encompassing eukaryotic algae and protozoa, are central actors in responding to and mediating these changes due to their pivotal ecological roles. They are not only major primary producers (e.g., diatoms, chrysophytes) fueling the food web but also key consumers through bacterivory and algaeivory, effectively channeling carbon and energy to higher trophic levels through the microbial loop [9]. Furthermore, by selectively grazing on bacteria, protists can regulate bacterial community composition and organic matter decomposition pathways, thereby indirectly influencing biogeochemical cycles such as carbon and nutrient regeneration [10]. Their short generation times and rapid physiological responses make them sensitive bioindicators of environmental change in lakes. Previous studies have demonstrated that protistan communities can exhibit resilience to seasonal freezing, often recovering quickly once conditions stabilize. In contrast, our understanding of protistan community dynamics during the critical freeze–thaw transition remains fragmented. Most research has focused on either the ice-covered period or the open-water season, treating them as discrete states, or has concentrated on bacterial communities [11]. A significant knowledge gap exists regarding the continuous succession of protistan communities across the complete ice-covered to ice-free transition, particularly along the vertical depth gradient which experiences the aforementioned environmental gradients (e.g., light, oxygen, nutrients) most acutely. Specifically, it is unclear how the vertical structure of the protistan community dismantles under ice and reassembles during thaw, and which environmental drivers are most critical at different depths. Addressing this gap is essential for predicting how climate-driven changes in ice phenology will affect the structure and function of lake ecosystems.

The Ningwu subalpine lake group, situated on the northern margin of the East Asian monsoon region [12], provides an ideal model system to investigate these questions. Among these lakes, Gonghai Lake is scientifically valuable for this study due to its distinct characteristics: (1) as the highest-altitude permanent freshwater lake in the group, it experiences a pronounced and prolonged seasonal freeze–thaw cycle (typically ice-covered from November to late March), amplifying the environmental contrasts between periods; (2) its location in a climate-sensitive zone makes it a potential sentinel for understanding the impacts of regional climate change on cold-region lakes; and (3) the relatively small size and well-defined hydrology help minimize confounding spatial heterogeneity, allowing a clearer focus on temporal and vertical dynamics. Here, we tracked the physicochemical parameters and protistan community structure at multiple depths in Gonghai Lake across the winter-spring transition. Based on the established links between freeze–thaw limnology and microbial ecology, we formulated the following hypotheses: (1) The composition and diversity of protistan communities will exhibit significant vertical stratification during the ice-covered period but will become more homogenized following ice-off and spring mixing. (2) The key environmental drivers of community composition will shift from factors like light availability and chemical stratification (under ice) to mixing and nutrient pulses (during thaw). This study aimed to address the following questions: (1) How did the composition and structure of the protistan community vary with depth during the transition from ice-covered to ice-free conditions in a subalpine lake? (2) What were the principal environmental factors driving changes in protistan community composition and diversity throughout the freeze–thaw process?

## 2. Materials and Methods

### 2.1. Site Description and Sampling

**Figure 1:**
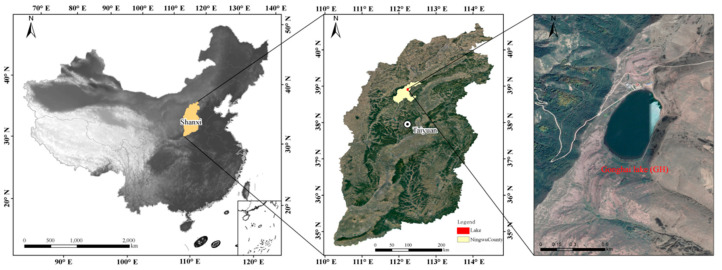
Map depicting Gonghai Lake (GH), a subalpine lake in Ningwu County, Shanxi Province, China, and the sampling location.

Gonghai Lake (GH) (38.91° N, 112.23° E) is located at the foot of the Guancen Mountains in Ningwu County, Shanxi Province, China, on the margin of the East Asian monsoon region (Figure 1). The lake lies at an altitude of 1,854 m above sea level, with a surface area of approximately 0.36 km^2^ and a maximum water depth of about 8 m. This area experiences a temperate monsoon climate characterized by cold, prolonged, and snowy winters, cool summers, and abundant precipitation. Water sampling was conducted on two occasions at the lake’s center: in December 2018 (representing the stable ice-covered period) and in April 2019 (representing the ice-free period immediately following the spring freeze–thaw cycle). During the ice-covered period, a manually operated ice auger was used to drill a sampling hole (diameter ~15 cm) through the ice cover. Any ice fragments and snow were thoroughly removed from the hole before sampling to prevent contamination. Throughout both sampling periods, a Plexiglass^®^ water sampler (LB-800, Qingdao, China) was used to collect water samples independently at 0, 2, 4, 6, and 8 m depths. To ensure representativeness, triplicate samples (1 L each) were collected at each depth during each of the two seasonal sampling campaigns. In total, 30 water samples were collected for biological and chemical analysis. At each sampling point, 3 L of water was collected. Of this, 2.5 L was filtered on-site through 0.2 μm polycarbonate membrane filters (Millipore, Jinteng, Tianjin, China) to collect aquatic microorganisms. The filter membrane with attached microorganisms was placed in a sterile centrifuge tube and stored in a portable liquid nitrogen container. The remaining 0.5 L water sample was placed in a vehicle-mounted refrigerator. After transportation to the laboratory, the centrifuge tubes containing the filter membranes were immediately transferred to a −20 °C freezer until DNA extraction. The water samples were stored in a 4 °C refrigerator until physicochemical parameter analysis was completed.

### 2.2. Physicochemical Analysis

The physicochemical parameters of the water samples were analyzed as follows. Temperature (T), pH, dissolved oxygen (DO), electric conductivity (EC), nitrate (NO_3_^−^) and ammonium (NH_4_^+^) were measured in situ using a portable multiparameter water quality monitor (Aquread AP-5000, Kent County, UK) according to the manufacturer’s calibration and measurement protocols. In the laboratory, total nitrogen (TN), nitrite (NO_2_^−^), sulfate (SO_4_^2−^) and phosphate (PO_4_^3−^) were determined using an automated discrete analyzer (CleverChem380, DeChem-Tech, Hamburg, Germany) following standard colorimetric methods. Meanwhile, total carbon (TC), total organic carbon (TOC) and inorganic carbon (IC) content were analyzed with a TOC analyzer (Shimadzu, TOC-V_CPH_, Shimane, Kyoto, Japan) in accordance with its operation manual (available at: https://max.book118.com accessed on 5 December 2025).

### 2.3. DNA Extractions, PCR Amplification, and High-Throughput Sequencing

Environmental DNA (eDNA) was extracted from the filters using the FastDNA SPIN Kit (MP Biomedicals, Solon, OH, USA). The concentration and purity of the extracted DNA were assessed with a Nanodrop One spectrophotometer (Thermo Scientific, Shanghai, China), and the eDNA was stored at −20 °C until further analysis. The V4 region of the 18S rRNA gene was amplified by PCR using the universal eukaryotic primers TAReuk454WD1F (5′-CCAGCASCYGCGGTAATTCC-3′) and TAReukEV3R (5′-ACTTTCGTTCTTGATYRA-3′) [13,14]. The amplification protocol followed the procedure described by Stoeck et al. [14]. The resulting amplicons were sequenced on the Illumina MiSeq PE300 platform. The sequencing libraries were prepared using the NEXTflex™ Rapid DNA-Seq Kit (Bluescape Scientific Co., Ltd., Beijing, China). Sequencing was performed using two-end chemistry. The sequencing run produced reads with a maximum length of 2 × 300 base pairs (Majorbio Bio-Pharm Technology Co., Ltd., Shanghai, China). Raw sequencing reads were processed as follows: quality filtering and adapter removal were performed with fastp (version 0.20.0), and paired-end reads were merged using FLASH (version 1.2.7). Low-quality sequences were removed based on the following criteria: (1) length < 150 bp; (2) average Phred score < 20; (3) presence of ambiguous nucleotides; and (4) mononucleotide repeats > 8 bp. After quality control, the remaining high-quality sequences from all 30 samples (totaling 2,145,910 reads) were clustered into operational taxonomic units (OTUs) at 97% similarity threshold using UPARSE (version 7.1). Chimeric sequences were identified and removed during this process. Representative sequences from each OTU were taxonomically classified by BLAST (version 2.13.0) search against the Protist_PR2_v4.13 database [15], with a minimum alignment identity of 70%. This yielded a final dataset of 1,019,657 protistan reads. An OTU table was constructed to record the abundance and taxonomy of each OTU and a total of 3859 OTUs were generated. Non-protistan sequences (including those from Fungi, Metazoa, Streptophyta, and unclassified groups at higher taxonomic levels) were removed; finally, 2479 protistan OTUs were retained for final ecological analysis. To standardize sequencing depth across samples, the OTU table was rarefied to the minimum number of quality-filtered reads per sample (minimum reads = 41,272).

### 2.4. Nucleic Acid Sequences

The sequence data of 18S rDNA genes were submitted to the NCBI GenBank as accession number SRP301277.

### 2.5. Data Analysis

The alpha diversity of the protistan community in each habitat was evaluated using observed OTUs, the Shannon index, and the Simpson index. The normality of the alpha diversity data was confirmed using the Shapiro–Wilk test. Differences in physicochemical factors, abundance of dominant taxa, and alpha diversity indices across seasons and depths were assessed by one-way ANOVA, followed by Tukey’s HSD post hoc tests for multiple comparisons. All the above analyses were conducted using SPSS 20.0 (IBM Corp., Armonk, NY, USA). The influence of environmental factors on dominant protistan phyla was visualized using redundancy analysis (RDA) in Canoco for Windows version 5.0. The co-occurrence network between environmental factors and the dominant protist orders/genera was constructed based on correlation analysis. Pairwise correlations were calculated using Spearman’s rank correlation method. Only robust and statistically significant correlations (|r| > 0.6, *p*-value < 0.05) were retained to define connections (edges) in the network. The resulting adjacency matrix was imported into the igraph package within R 4.3.1 for network construction and topological analysis. Key topological metrics, including the average degree, average path length, clustering coefficient, and modularity, were computed to characterize the overall network structure. Finally, the network was visualized using Gephi (version 0.9.7) for graphical representation. Beta diversity, based on Bray–Curtis distances, was analyzed to examine structural variations in protistan communities between seasons and across the five depths. ANOSIM was used to test for significant differences in community structure among seasons and depths, and the results were visualized using principal coordinates analysis (PCoA) in Canoco for Windows version 5.0. The impacts of environmental factors and sampling depth on community structure were further assessed using canonical correspondence analysis (CCA) and redundancy analysis (RDA). Environmental factors were initially selected through stepwise regression and the Monte Carlo permutation test (Supplementary Table S1). Only factors with a variance inflation factor (VIF) < 10 were retained in the final CCA and RDA models. A significance level of *p* < 0.05 was applied for all statistical tests.

## 3. Results

### 3.1. Shifts in the Vertical Stratification of Lake Water Physicochemistry Between Ice-Covered and Ice-Free Periods

The seasonal freeze–thaw process led to significant variations in the physicochemical parameters of the lake water. Parameters such as T, DO, NO_3_^−^, NO_2_^−^, TC, TOC, and IC concentration were significantly higher after ice melt, whereas EC and NH_4_^+^ concentrations were greater under the ice cover in winter (*p* < 0.05) (Table 1). Although the lake has a maximum depth of only 8 m, it exhibits clear seasonal physicochemical stratification, particularly during the prolonged ice-covered period. Under the ice cover, key parameters such as water temperature and nutrients (NO_3_^−^) showed significant variation with depth during our winter sampling. This stratification creates distinct ecological niches along the depth gradient. The ice-free period is characterized by thermal homogeneity and wind-driven mixing, leading to a more uniform vertical distribution of physicochemical parameters (Supplementary Figure S1).

### 3.2. Changes in Protistan Community Composition Across Seasonal Freeze–Thaw Cycles and Lake Depths

#### 3.2.1. Protistan Community Composition Across Seasonal Freeze–Thaw

**Figure 2:**
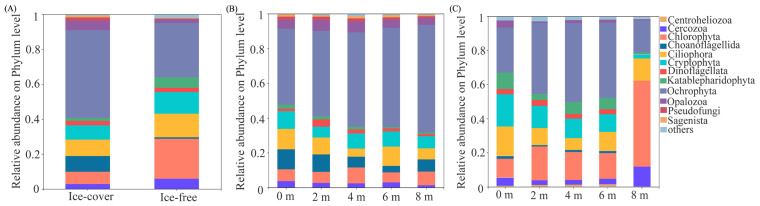
Relative abundance of dominant protistan phyla (average > 1%) across different periods: (**A**) comparison between ice-covered and ice-free conditions, (**B**) variations across depths during the ice-covered period, and (**C**) variations across depths during the ice-free period.

**Figure 3:**
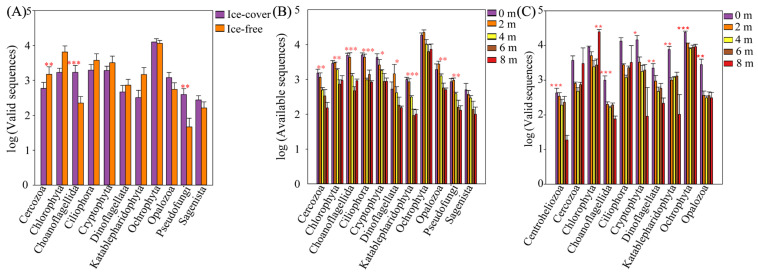
Differences in dominant phyla abundance during (**A**) both periods, the (**B**) ice-covered period and the (**C**) ice-free period. The differences between different seasons and different depths were analyzed by one-way ANOVA * *p* < 0.05, ** *p* < 0.01 and *** *p* < 0.001.

Taxa with a relative abundance exceeding 1% were classified as dominant; those below this threshold were grouped as “others”. During the ice-covered winter period, the protistan community comprised 11 dominant phyla. Ochrophyta was the most abundant (50.21%), followed by Ciliophora (9.42%), Choanoflagellida (8.99%), Cryptophyta (8.18%), Chlorophyta (7.03%), Opalozoa (5.38%), Cercozoa (2.85%), Dinoflagellata (2.50%), Pseudofungi (1.88%), Katablepharidophyta (1.77%), and Sagenista (1.15%). During the ice-free spring, the community consisted of 8 dominant phyla. Ochrophyta remained dominant (31.08%), followed by Chlorophyta (22.86%), Ciliophora (13.50%), Cryptophyta (12.40%), Katablepharidophyta (6.01%), Cercozoa (5.93%), Dinoflagellata (2.45%), and Opalozoa (2.28%) (Figure 2A). Cercozoa exhibited significantly greater abundance in the ice-free period, while Choanoflagellida and Pseudofungi were more abundant in the ice-covered period (Figure 3).

At the order level, among the 21 dominant orders, Bacillariophyta (43.77%) was the most abundant in winter, whereas Dictyochophyceae (14.24%) dominated in spring (Supplementary Figure S2A). At the genus level, *Stephanodiscus* (39.18%) showed the highest relative abundance in winter, while *Pedinellales* (13.97%) was the dominant genus in spring (Supplementary Figure S2A,B). At finer taxonomic resolutions, the abundances of 12 out of 21 dominant orders and 18 out of 28 dominant genera also showed significant seasonal variations (Supplementary Figure S3).

#### 3.2.2. Protistan Community Composition Across Different Depths

During the ice-covered period, the community was dominated by Ochrophyta (43.67–62.05%), whose abundance increased with depth (Figure 2B). Other dominant phyla mostly showed surface-enriched patterns, with significant depth variation for all but Ochrophyta and Sagenista (Figure 3B). This Ochrophyte dominance was attributed to diatoms (class Bacillariophyceae), particularly the class Mediophyceae (33.95–53.23% relative abundance). The most abundant genus within Bacillariales was *Stephanodiscus* (30.39–47.16%). The relative abundances of Mediophyceae and *Stephanodiscus* peaked at 4 m depth (Supplementary Figures S2A–D and S3C,D). During the ice-free period, Ochrophyta (28.63–46.35%) was dominant only to 6 m, being replaced by Chlorophyta at 8 m (50.03%; Figure 2C). Only Cercozoa and Ciliophora showed no significant depth variation at the phylum level (Figure 3C). However, significant variation with depth was common for finer taxa, affecting 18 orders and 25 genera (Supplementary Figures S2E,F and S3E,F).

#### 3.2.3. Relationship Between Dominant Taxa and Environmental Factors

**Figure 4:**
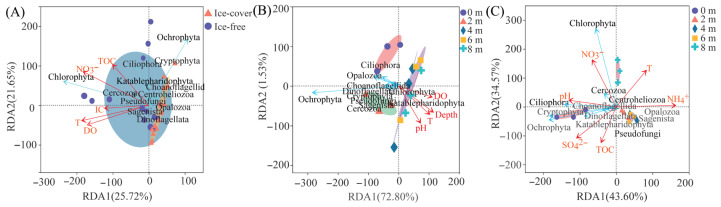
Redundancy analysis (RDA) of dominant protistan phyla and environmental variables. (**A**) Comparison of the ice-covered and ice-free periods. (**B**) Variation across the five sampling depths during the ice-covered period. (**C**) Variation across the five sampling depths during the ice-free period. Only environmental variables with a variance inflation factor (VIF) < 10 and showing a significant fit to the ordination are displayed. Ellipses represent the 95% confidence intervals around the centroid of each sample group, based on the standard error of the point scores.

Redundancy analysis (RDA) was performed to identify the key environmental factors shaping the protistan community composition at the phylum level. The analysis revealed that TOC, NO_3_^−^, IC, T and DO were significant predictors of seasonal variation in the dominant phyla (*p* < 0.05) (Figure 4A). The significant environmental drivers differed between the two periods. During the ice-covered period, community composition was primarily associated with DO, depth, T, and pH (Figure 4B). In contrast, during the ice-free period, it was significantly influenced by NO_3_^−^, pH, SO_4_^2−^, TOC, NH_4_^+^ and T (Figure 4C). At finer taxonomic levels, TOC was the key predictor for the variation in dominant orders across seasons, while SO_4_^2−^ was the main factor for dominant genera (Supplementary Figure S4A,B and Table S4). During the ice-covered period, DO was the most important factor structuring both dominant orders and genera (Supplementary Figure S4C,D and Table S4). In the ice-free period, SO_4_^2−^ emerged as the predominant factor influencing the dominant taxa at both order and genus levels (Supplementary Figure S4E,F and Table S4).

### 3.3. Dynamics and Drivers of the Protistan Community Diversity Across Seasons and Depths

**Figure 5:**
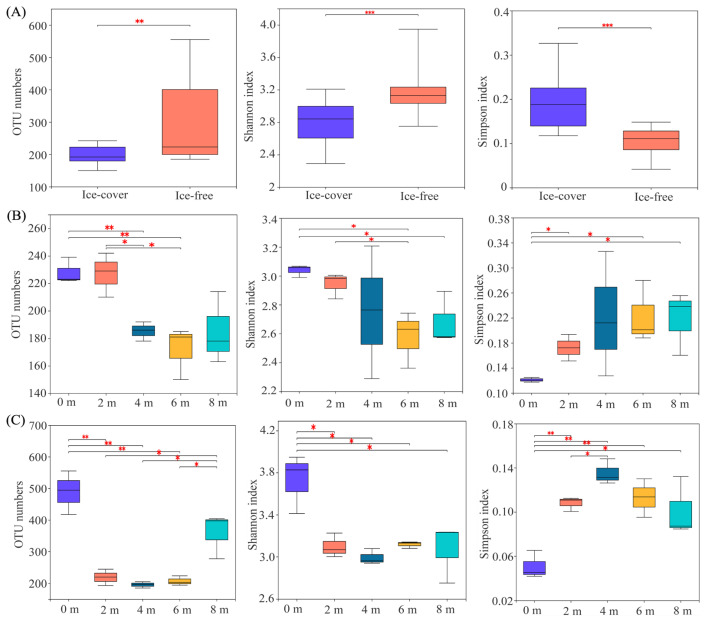
Variations in protistan community alpha diversity. (**A**) Diversity indices across seasonal periods (ice-covered vs. ice-free). (**B**) Depth-related variations during the ice-covered period. (**C**) Depth-related variations during the ice-free period. Significant differences between samples were determined using one-way ANOVA, * *p* < 0.05, ** *p* < 0.01, *** *p* < 0.001.

The transition from the ice-covered to the ice-free period was accompanied by significant shifts in alpha diversity. Both the observed OTU number and Shannon index increased significantly (*p* < 0.05), from 199.47 ± 13.62 to 293.73 ± 35.42 and from 2.80 ± 0.11 to 3.20 ± 0.15, respectively. In contrast, the Simpson index decreased significantly from 0.19 ± 0.01 to 0.10 ± 0.01 (Figure 5). Alpha diversity also varied with sampling depth, exhibiting distinct patterns between the two periods. During the ice-covered period, the highest OTU numbers and Shannon indices were observed in the upper water layers (0 m and 2 m), and their values decreased gradually with increasing depth. Conversely, the Simpson index was highest in the deeper layers (6 m and 8 m) (Figure 5). In the ice-free period, the OTU numbers were greater at the surface (0 m) and bottom (8 m) than in the mid-depth layers (2 m, 4 m, 6 m). The Shannon index peaked at the surface, while the Simpson index was highest at 4 m (Figure 5).

**Figure 6:**
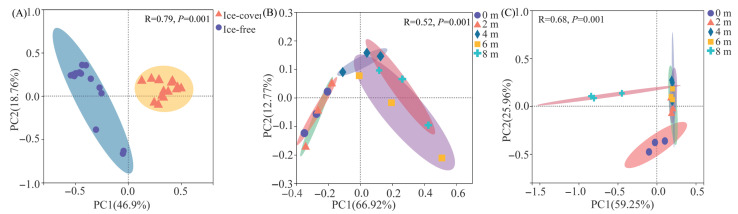
Principal Coordinate Analysis (PCoA) of protistan community structure based on Bray–Curtis dissimilarity at the OTU level. (**A**) Separation between the ice-covered and ice-free periods. (**B**) Variation across the five sampling depths during the ice-covered period. (**C**) Variation across the five sampling depths during the ice-free period. The results of the ANOSIM test (R statistic) for pairwise comparisons are displayed on the respective plots. Ellipses represent the 95% confidence intervals around the centroid of each sample group, based on the standard error of the point scores.

Protistan community structure, assessed by PCoA based on Bray–Curtis distances, formed distinct clusters according to season and depth (Figure 6). ANOSIM confirmed that the observed differences between seasons and among depths within each season were statistically significant (*p* < 0.05) (Figure 6).

**Figure 7:**
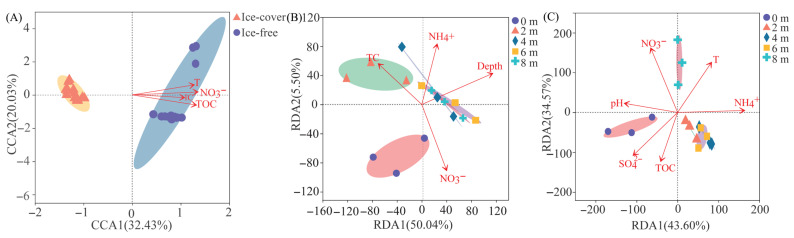
Key environmental drivers of protistan community structure identified by RDA or CCA on OTU level. (**A**) Drivers of seasonal variation. (**B**) Drivers across depths under ice-covered conditions. (**C**) Drivers across depths under ice-free conditions. Ellipses represent the 95% confidence intervals around the centroid of each sample group, based on the standard error of the point scores.

Redundancy Analysis and Canonical Correspondence Analysis revealed distinct key drivers of the protistan community under different conditions. TOC was the strongest predictor of seasonal variation (R^2^ = 0.897, *p <* 0.01) had the largest explanation (Figure 7A, Table 2). During the ice-covered period, sampling depth was the most influential factor across depths (R^2^ = 0.699, *p* < 0.01) (Figure 7B, Table 2), whereas NO_3_^−^ was the primary driver during the ice-free period (R^2^= 0.738, *p* < 0.01) (Figure 7C, Table 2).

## 4. Discussion

### 4.1. Seasonal Shift: From Physically Driven to Resource-Driven Community Assembly

Protists form the foundational level of aquatic food webs [16]. In seasonally ice-covered lakes, the annual freeze–thaw cycle imposes a radical regime shift on ecosystem processes, fundamentally altering the template for community assembly [17,18]. Our study tested and confirmed two hypotheses: (1) protistan communities transition from vertically stratified structures under ice to more homogenized states after ice-off, and (2) the primary environmental drivers shift from factors linked to physical stratification to those related to resource supply and mixing.

The ice-covered period was characterized by a strong, niche-based vertical structure. The increasing dominance of Ochrophytes with depth, culminating in a distinct peak of *Stephanodiscus* at 4 m (Figure 2, Supplementary Figure S2), reflects a biological response to a steep vertical gradient in light and chemical conditions under a stable water column [19], rather than a mere statistical correlation with depth. Although Gonghai Lake has a maximum depth of only 8 m, the absence of wind-driven turbulence under ice allowed for the development of pronounced stratification, as evidenced by the marked change in T and NO_3_^−^ from surface to bottom (Supplementary Figure S1). The dominance of *Stephanodiscus*, many of which possess physiological adaptations to low-light and low-temperature conditions (e.g., membrane lipid modifications) [20], is a classic adaptation to the stable and low-light hypolimnetic niches of ice-covered lakes [6,21,22].

The ice-off period represents a switch to a resource-driven assembly paradigm [18]. Increased wind mixing homogenized the water column, broke down the vertical chemical gradients and made resources like NO_3_^−^ and TOC more uniformly accessible—though their concentrations varied primarily due to external inputs [23]. Consequently, community structure became less predictable by vertical position alone and more tightly coupled to the dynamics of these newly available resources [24], as shown by the strong association with NO_3_^−^ and TOC in our results (Figure 7). The shift in bottom-layer dominance from Ochrophyta to Chlorophyta (Figure 2C) likely reflects this change: chlorophytes often exhibit higher growth rates and competitive advantage under higher light and temperature conditions post-thaw, and their surge may have been fueled by nutrient pulses from mixing and runoff [25,26,27].

### 4.2. Mechanistic Links Between Environmental Filters and Taxonomic Succession

The multidimensional taxonomic shift from phylum to genus is a direct manifestation of environmental filtering operating at different intensities across seasons [28]. The ice-covered period acted as a strong filter, selecting for taxa with traits conferring fitness in a cold, dark, and stratified environment [29]. This explains not only the dominance of Cryotolerant diatoms but also the prevalence of heterotrophic Choanoflagellates and other Stramenopiles (Figure 3, Supplementary Figure S2), which may have thrived on bacterial production sustained by the degradation of organic matter accumulating in the isolated hypolimnion [30,31]. The significant increase in alpha diversity after ice melt (Figure 5) aligns with the relaxation of this harsh environmental filter [32]. The reintroduction of light, atmospheric gas exchange, and allochthonous nutrient and carbon subsidies (Supplementary Figure S1) created a broader spectrum of ecological niches [33,34]. This supported a more diverse assemblage, including light-demanding autotrophs (e.g., Cryptomonas, Chlamydomonas) and the heterotrophic protists that prey upon the burgeoning bacterial community fueled by terrestrial organic carbon [35]. The central role of TOC and NO_3_^−^ in explaining seasonal community variation (Table 1) underscores the shift to a bottom-up, resource-controlled system during the ice-free period, consistent with findings in other mountain lakes receiving substantial catchment inputs [30,31].

### 4.3. Evaluating the Role of Depth and Vertical Gradients

In a shallow, seasonally stratified lake such as Gonghai, depth is not the fundamental ecological driver. Instead, it functions as a master variable that integrates and reflects the vertically structured physicochemical landscape, which in turn directly governs biological processes [36]. Under ice cover, the stabilized water column allowed for the development of steep vertical gradients. The availability of photosynthetically active radiation attenuated sharply with depth. Concurrently, DO concentrations created a pronounced gradient, with higher levels near the ice-water interface and lower levels in the hypolimnion [37]. The observed peak abundance of *Stephanodiscus* at 4 m (Supplementary Figure S2) likely represents a vertical optimum niche, balancing sufficient light for photosynthesis against the risks of near-surface freezing and near-bottom anoxia, while positioning itself to access nutrients diffusing from sediments [38]. In contrast, during the well-mixed ice-free period, wind-driven turbulence homogenized the water column, and depth lost its predictive power as a proxy for strong chemical gradients. However, community structure differences persisted between the bottom (8 m) and shallower depths (Figure 6). This pattern can be attributed to benthic–pelagic coupling [39]. The sediment–water interface acts as a distinct ecotone of intense biogeochemical activity. Nutrient fluxes from the sediment created a localized zone of elevated resource availability [40], sustaining a distinct “benthic influence zone” community. This subtle yet critical pattern underscores the necessity of depth-resolved sampling.

## 5. Conclusions

This study demonstrated that the seasonal freeze–thaw fundamentally reorganized protistan communities in a shallow mountain lake. The results confirmed that communities were vertically stratified under ice but became more homogenized after ice-off, and the dominant environmental drivers shifted from stratification-related factors to those associated with resource availability and mixing. Taxa such as *Stephanodiscus* and the post-thaw rise in Chlorophyta served as compelling examples of this regime shift. While this work empirically revealed pronounced heterogeneity under ice cover and underscored the critical importance of high-resolution sampling, we note that the demonstrated correlations require future experimental studies to establish mechanistic causality. The changes in ice phenology may alter these delicate vertical structures, with potential consequences for overall ecosystem function.

## Figures and Tables

**Table 1:** Physicochemical parameters in ice-covered and ice-free periods of Gonghai Lake.

Factors	Ice-Covered	Ice-Free
T (°C)	3.60 ± 0.74 b	6.17 ± 1.26 a
pH	7.94 ± 0.05 b	8.69 ± 0.10 a
DO (mg·L^−1^)	8.90 ± 0.96 b	11.23 ± 0.75 a
EC (μS·cm^−1^)	963.27 ± 14.15 a	826.13 ± 18.97 b
NO_3_^−^ (mg·L^−1^)	0.01 ± 0.01 b	0.03 ± 0.01 a
NO_2_^−^ (mg·L^−1^)	0.02 ± 0.01 b	0.03 ± 0.02 a
NH_4_^+^ (mg·L^−1^)	4.21 ± 0.18 a	0.11 ± 0.06 b
TC (mg·L^−1^)	119.53 ± 1.92 b	131.62 ± 1.09 a
TOC (mg·L^−1^)	18.94 ± 2.31 b	29.14 ± 1.34 a
IC (mg·L^−1^)	100.60 ± 1.56 b	102.47 ± 0.66 a
SO_4_^2−^ (mg·L^−1^)	19.72 ± 5.08 a	20.23 ± 2.55 a
PO_4_^3−^ (mg·L^−1^)	0.45 ± 0.38 a	0.25 ± 0.16 a

Note: The data were shown as the means ± standard error, and different lowercase letters represent significant differences (*p* < 0.05). Abbreviations: T represents temperature; DO represents dissolved oxygen; EC represents electroconductibility; TN represents total nitrogen; NO_3_^−^ represents nitrate; NO_2_^−^ represents nitrite; NH_4_^+^ represents ammonium; TC represents total carbon; IC represents inorganic carbon; TOC represents organic carbon; SO_4_^2−^ represents sulfate and PO_4_^3−^ represents phosphate. Significant differences between samples were determined using one-way ANOVA at *p* < 0.05 and different letters indicate significant differences.

**Table 2:** Explanatory power of environmental parameters on the first two RDA and CCA axes.

Between Two Periods					Ice-Covered Period					Ice-Free Period				
Factors	CCA1	CCA2	R^2^	*p*	Factors	RDA1	RDA2	R^2^	*p*	Factors	RDA1	RDA2	R^2^	*p*
TOC	0.951	−0.309	0.897	0.001	Depth	0.816	0.578	0.699	0.001	NO_3_^−^	−0.3771	0.926	0.738	0.002
T	0.955	0.297	0.831	0.001	NO_3_^−^	0.153	−0.988	0.449	0.035	NH_4_^+^	0.999	0.042	0.674	0.002
NO_3_^−^	0.997	0.076	0.821	0.001	NH_4_^+^	0.337	0.942	0.439	0.040	SO_4_^2−^	−0.7195	−0.694	0.584	0.004
IC	0.998	−0.058	0.518	0.002	TC	−0.668	0.745	0.289	0.045	T	0.566	0.824	0.561	0.007
-	-	-	-	-	-	-	-	-	-	pH	−0.9878	0.156	0.429	0.033
-	-	-	-	-	-	-	-	-	-	TOC	−0.3361	−0.942	0.408	0.038

Abbreviations: T represents temperature; NO_3_^−^ represents nitrate; NH_4_^+^ represents ammonium; TC represents total carbon; IC represents inorganic carbon; TOC represents organic carbon; SO_4_^2−^ represents sulfate; - represents no value.

## Data Availability

The original contributions presented in this study are included in the article. Further inquiries can be directed to the corresponding author.

## Supplementary Materials

The following supporting information can be downloaded at: https://www.mdpi.com/article/10.3390/microorganisms13122869/s1, Figure S1: The variation trend of water physicochemical parameters with sampling depth in the ice-covered and ice-free periods. The green letters represent the difference in depths during the ice-covered period, the red letters represent the difference in depths during the ice-free period, and different letters represent significant differences; Figure S2: Relative abundance of the dominant orders and dominant genera (with average relative abundance > 1%) (**A**,**B**) between two periods and (**C**,**D**) among five different depths in the ice-covered period and (**E**,**F**) among five different depths in the ice-free period; Figure S3: Difference among dominant orders and dominant genera for (**A**,**B**) between two periods; (**C**,**D**) among five different depths in the ice-covered period and (**E**,**F**) among five different depths in the ice-free period. The differences between seasons and depths were analyzed by one-way ANOVA, * *p* < 0.05, ** *p* < 0.01 and *** *p* < 0.001; Figure S4: Network diagram showing Spearman correlations between dominant orders and dominant genera and physicochemical variables for (**A**,**B**) between two periods, (**C**,**D**) among five different depths in the ice-covered period and (**E**,**F**) among five different depths in the ice-free period. Pairwise correlations without assigned color represent correlations that are not significant; Table S1: Variance inflation factor (VIF) selection results of 12 environment parameters; VIF values less than 10 were considered to have little collinearity with each other; Table S2: Mantel test showing the correlation between protistan community alpha diversity and environment parameters in this study; Table S3: 2018–2019 Annual precipitation/mm; Table S4: Environmental factors in network center coefficient.
