# Supplementary material for: Seasonal Shifts from Water Depth to Nitrate Reorganize Protistan Communities Following Lake Freeze–Thaw Events
Source: Microorganisms. 2025 Dec 17;13(12):2869. doi: 10.3390/microorganisms13122869 (PMC12736319; doi:10.3390/microorganisms13122869)
Supplement: Supplementary file 1 [file microorganisms-13-02869-s001.zip › microorganisms-3994124-supplementary.pdf]

Supplementary Material

Supplementary Tables

Table S1. Variance Inflation Factor（VIF）selection results of 12 environment parameters, VIF values less than 10 were considered to have little collinearity with each other.

Factors	Between two periods		Frozen period		Ice free period	
	Before	After	Before	After	Before	After
T (°C)	24.79	**4.62**	93.42	-	103606.07	**4.41**
pH	92.56	-	1471.71	-	396.20	**1.40**
DO (mg·L⁻¹)	40.25	-	1090.03	-	331781.17	-
EC (μS·cm⁻¹)	329.95	-	3935.79	-	53421.03	-
NO_3^- (mg·L⁻¹)	10.44	**6.08**	4314.64	**2.39**	12791.28	**3.21**
NO_2^- (mg·L⁻¹)	2.26	**1.82**	1041.41	-	10094.82	**1.47**
NH_4^+ (mg·L⁻¹)	444.72	-	3481.28	**3.92**	32779.48	**4.35**
TC (mg·L⁻¹)	45616.43	-	4612792.25	**2.33**	3026085.96	**1.74**
TOC (mg·L⁻¹)	31545.33	**4.11**	6379829.39	-	5252013.64	-
IC (mg·L⁻¹)	3110.84	**1.84**	2967670.47	**2.37**	1116682.67	**1.30**
SO_4^{2-} (mg·L⁻¹)	123.49	-	69.82	**2.03**	37288.06	**3.33**
PO_4^{3-} (mg·L⁻¹)	3.00	**1.69**	139.71	**2.61**	1810.70	-

Abbreviations: T represents temperature; DO represents dissolved oxygen; EC represents electroconductibility; TN represents total nitrogen; NO_3^- represents nitrate; NO_2^- represents nitrite; NH_4^+ represents Ammonium; TC represents total carbon; IC represents inorganic carbon ; TOC represents organic carbon; SO_4^{2-} represents sulfate and PO_4^{3-} represents phosphate.

Table S2. Mantel test showing the correlation between protistan community alpha diversity and environment parameters in this study.

Factors	Alpha diversity index		
	OTUs	Shannon	Simpson
T (°C)	-0.031	-0.035	-0.017
pH	**0.269****	**0.198****	**0.225****
DO (mg·L⁻¹)	0.008	-0.025	0.018
EC (μS·cm⁻¹)	0.048	**0.097***	**0.153***
NO_3^- (mg·L⁻¹)	**0.187***	0.138	**0.318***
NO_2^- (mg·L⁻¹)	0.125	-0.003	-0.062
NH_4^+ (mg·L⁻¹)	0.086	**0.290****	**0.427****
TC (mg·L⁻¹)	0.038	**0.225****	**0.271****
TOC (mg·L⁻¹)	0.031	**0.244****	**0.320****
IC (mg·L⁻¹)	-0.028	0.039	0.154
SO_4^{2-} (mg·L⁻¹)	**0.125***	**0.206****	**0.285****
PO_4^{3-} (mg·L⁻¹)	0.019	0.164	**0.191***

*$p < 0.05$, **$p < 0.01$ and ***$p < 0.001$. Abbreviations: T represents temperature; DO represents dissolved oxygen; EC represents electroconductibility; TN represents total nitrogen; NO_3^- represents nitrate; NO_2^- represents nitrite; NH_4^+ represents Ammonium; TC represents total carbon; IC represents inorganic carbon ; TOC represents organic carbon; SO_4^{2-} represents sulfate and PO_4^{3-} represents phosphate.

Table S3. 2018-2019 Annual precipitation/mm.

Year	Jan	Feb	Mar	Apr	May	Jun	Jul	Aug	Sep	Oct	Nov	Dec
2018	13.7	13.1	9.8	30.8	21.4	70.2	175.5	105.3	48.1	53.2	5.8	9.4

2019	1	8.3	11.5	31	18.8	29.5	145.4	122.6	19.6	102.5	13.4	8.9

Table S4. Environmental factors in network center coefficient.

Period	Order level				Genus level			
	**Node name**	**Degree centrality**	**Closeness centrality**	**Betweenness centrality**	**Node name**	**Degree centrality**	**Closeness centrality**	**Betweenness centrality**
Between two periods	TOC	0.469	0.571	0.106	SO_4^{2-}	0.514	0.597	0.110
	SO_4^{2-}	0.469	0.571	0.103	TC	0.487	0.578	0.091
	TC	0.438	0.552	0.079	TOC	0.487	0.578	0.091
	NH_4^+	0.406	0.533	0.076	NH_4^+	0.432	0.544	0.062
	T	0.375	0.516	0.109	T	0.405	0.529	0.081
	EC	0.344	0.500	0.068	EC	0.405	0.529	0.081
	Depth	0.344	0.457	0.186	DO	0.378	0.514	0.055
	pH	0.281	0.432	0.020	pH	0.351	0.500	0.034
	DO	0.281	0.471	0.033	Depth	0.351	0.474	0.170
	NO_3^-	0.281	0.432	0.017	NO_3^-	0.270	0.463	0.015
	IC	0.156	0.390	0.002	IC	0.189	0.394	0.003
Ice- covered period	DO	0.657	0.700	0.392	DO	0.677	0.708	0.291
	T	0.600	0.583	0.133	T	0.647	0.654	0.177
	Depth	0.600	0.583	0.133	Depth	0.588	0.607	0.125
	EC	0.514	0.515	0.076	EC	0.559	0.586	0.109
	pH	0.486	0.500	0.067	pH	0.471	0.531	0.081
	TOC	0.086	0.343	0.002	NH_4^+	0.088	0.340	0.002
	NO_3^-	0.057	0.324	0.111	SO_4^{2-}	0.059	0.340	0.001
	IC	0.029	0.318	0.000	TOC	0.059	0.354	0.114
	TC	0.029	0.330	0.000	IC	0.029	0.213	0.000
	SO_4^{2-}	0.029	0.330	0.000	TC	0.029	0.333	0.000
	NH_4^+	0.029	0.324	0.000	NO_3^-	0.029	0.304	0.000
	NO^{2-}	0.029	0.201	0.000	-	-	-	-
Ice-free period	SO_4^{2-}	0.441	0.523	0.151	SO_4^{2-}	0.4762	0.532	0.1299
	Depth	0.412	0.508	0.127	Depth	0.4762	0.532	0.1382
	T	0.382	0.493	0.108	T	0.4048	0.4821	0.0933
	DO	0.382	0.453	0.081	DO	0.3571	0.417	0.0493
	PO_4^{3-}	0.382	0.493	0.394	PO_4^{3-}	0.3333	0.417	0.1376
	EC	0.206	0.391	0.020	EC	0.2857	0.406	0.0598
	pH	0.147	0.374	0.095	NH_4^+	0.1429	0.3149	0.0138
	TC	0.088	0.351	0.002	NO_3^-	0.119	0.119	0.0116
	TOC	0.088	0.351	0.002	pH	0.0952	0.3214	0.049
	NO_3^-	0.059	0.281	0.059	TOC	0.0952	0.3149	0.0015
	NH_4^+	0.059	0.286	0.000	TC	0.0476	0.3025	0.0002
	IC	0.029	0.306	0.000	IC	0.0238	0.2707	0

Abbreviations: T represents temperature; DO represents dissolved oxygen; EC represents electroconductibility; TN represents total nitrogen; NO_3^- represents nitrate; NO_2^- represents nitrite; NH_4^+ represents Ammonium; TC represents total carbon; IC represents inorganic carbon ; TOC represents organic carbon; SO_4^{2-} represents sulfate and PO_4^{3-} represents phosphate; - represents there is no value.

Supplementary Figures

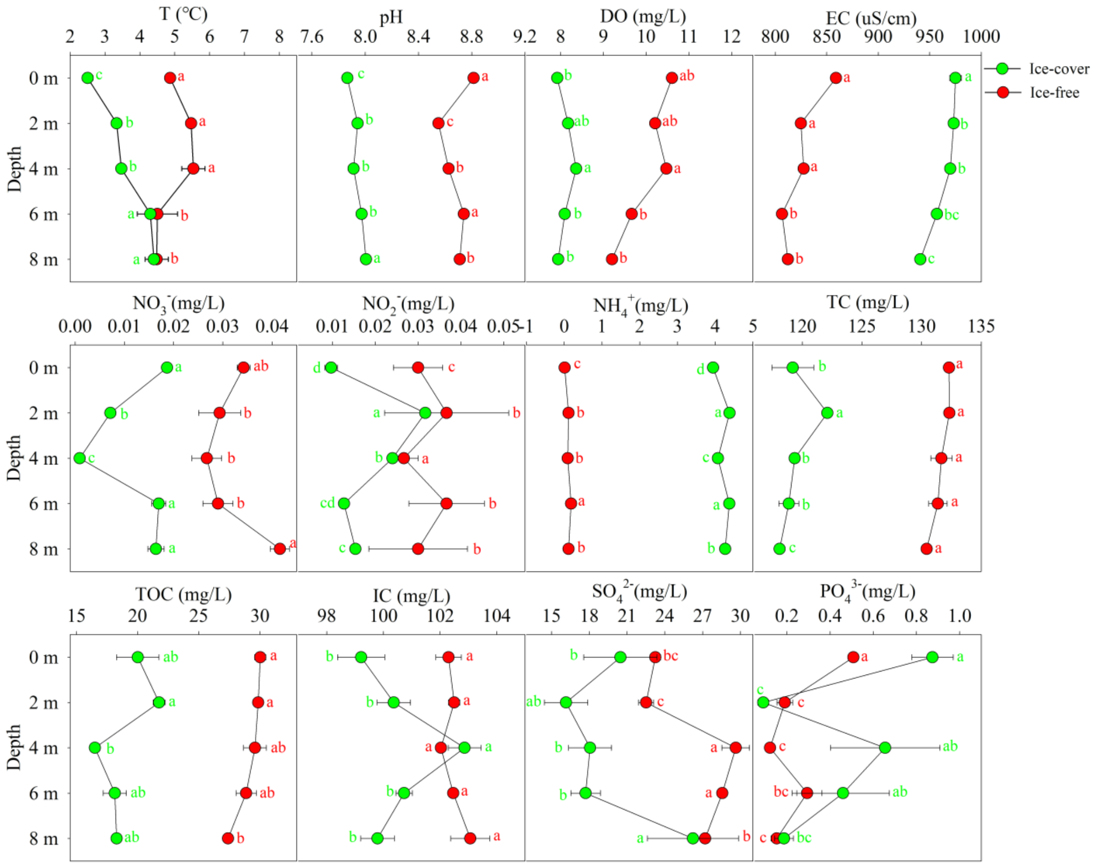

Figure S1. The variation trend of water physicochemical parameters with sampling depth in the ice-covered and ice-free periods. The green letters represent the difference of different depths during the ice-covered period, the red letters represent the difference of different depths during the ice- free period, and the different letters represent the significant difference. Abbreviations: T represents temperature; DO represents dissolved oxygen; EC represents electroconductibility; TN represents total nitrogen; NO_3^- represents nitrate; NO_2^- represents nitrite; NH_4^+ represents Ammonium; TC represents total carbon; IC represents inorganic carbon ; TOC represents organic carbon; SO_4^{2-} represents sulfate and PO_4^{3-} represents phosphate.

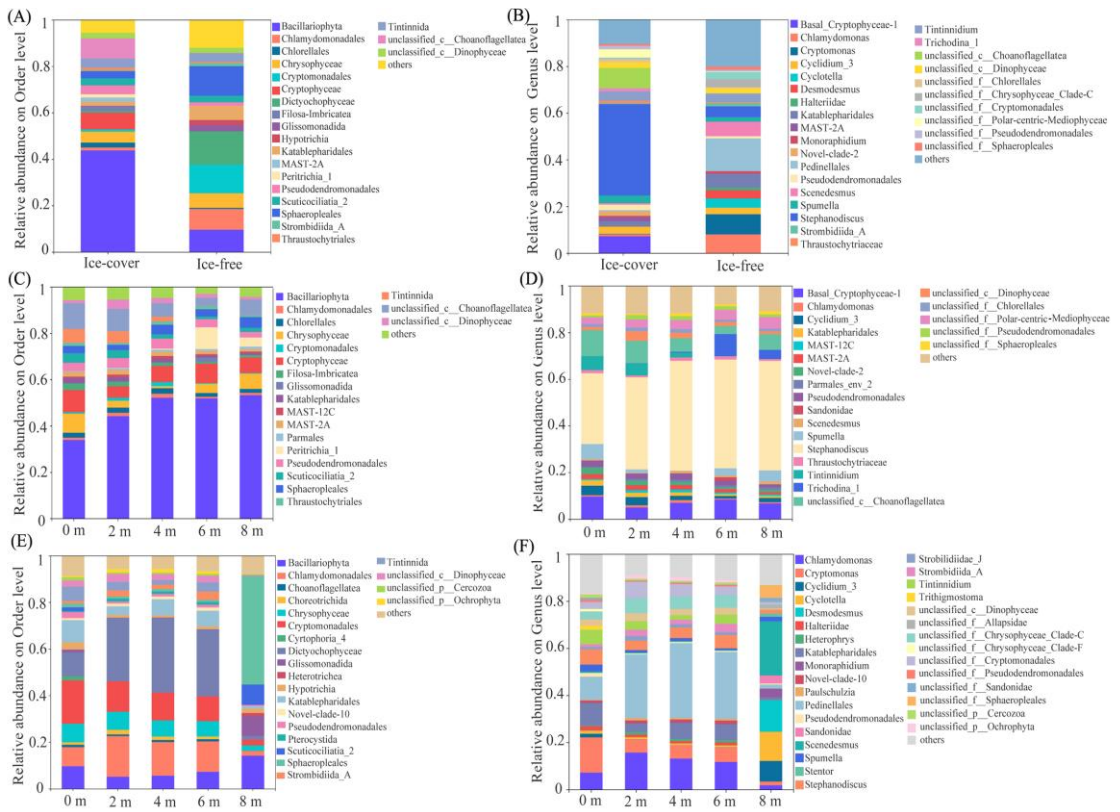

Figure S2. Relative abundance of the dominant orders and dominant genera (with average relative abundance > 1%) during the (A and B) between two periods and the (C and D) among five different depths of ice-covered period and the (E and F) among five different depths of ice-free period.

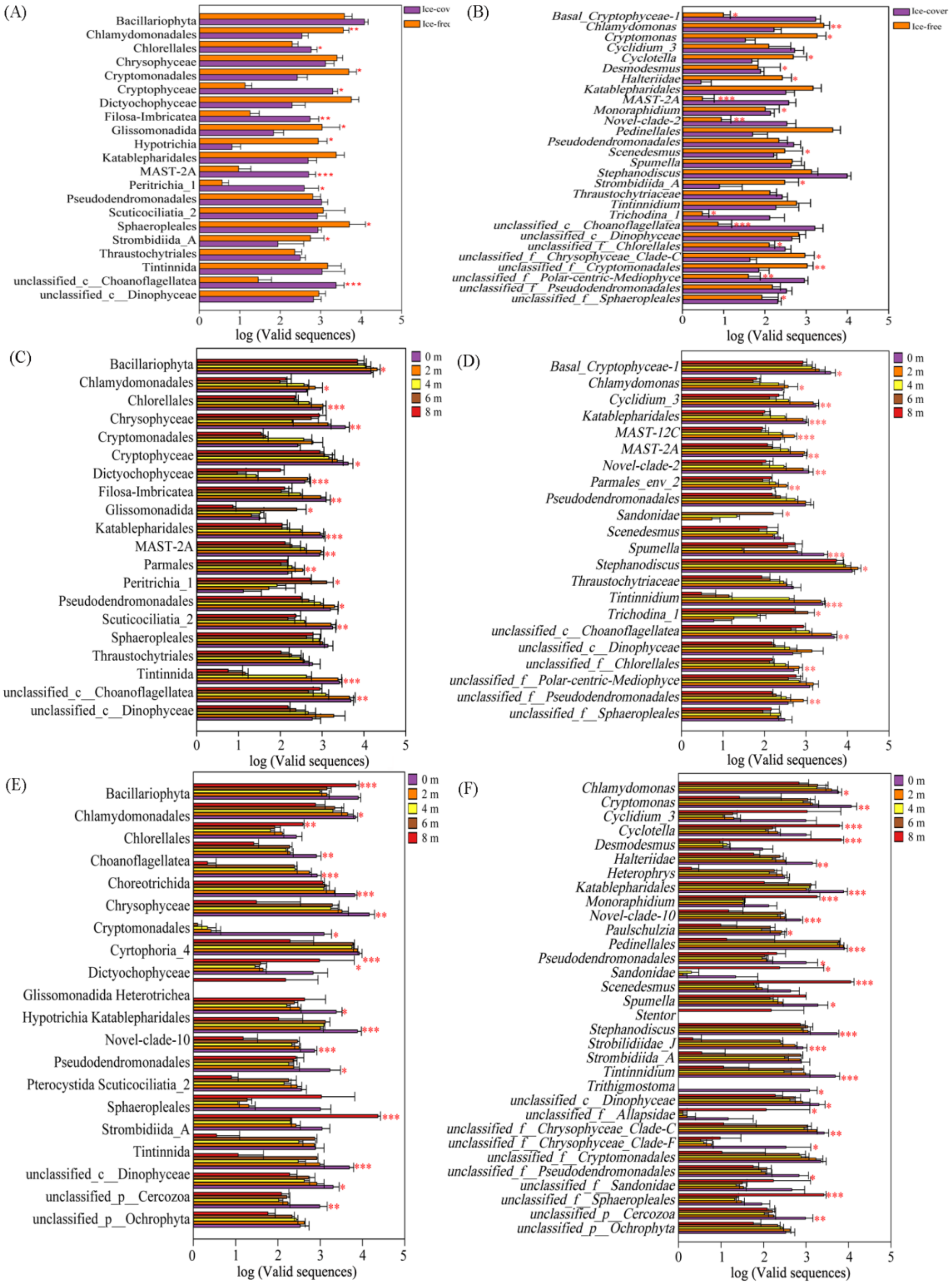

Figure S3. Difference among dominant orders and dominant genera for (A and B) between two periods; (C and D) among five different depths of ice-covered period and (E and F) among five different depths of ice-free period. The differences between different seasons and different depths were analyzed by one-way ANOVA, *$p < 0.05$, **$p < 0.01$ and ***$p < 0.001$.

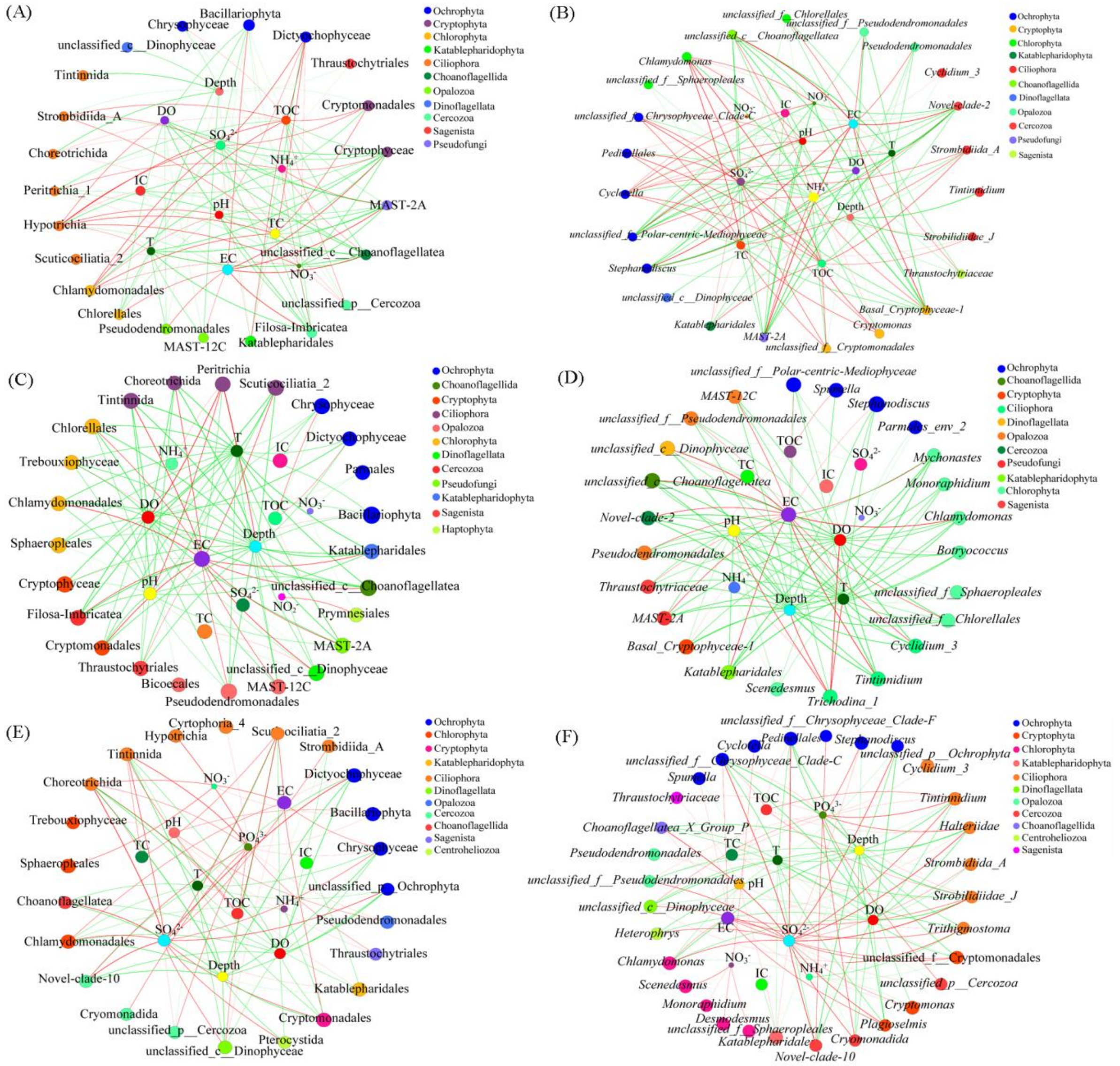

Figure S4. Network diagram showing Spearman correlations between dominant orders and dominant genera and physicochemical variables for (A and B) between two periods, (C and D) among five different depths of ice- covered period and (E and F) among five different depths of ice-free period. Pairwise correlations without assigned color represent correlations that are not significant. Abbreviations: T represents temperature; DO represents dissolved oxygen; EC represents electroconductibility; TN represents total nitrogen; NO_3^- represents nitrate; NO_2^- represents nitrite; NH_4^+ represents Ammonium; TC represents total carbon; IC represents inorganic carbon ; TOC represents organic carbon; SO_4^{2-} represents sulfate and PO_4^{3-} represents phosphate.
